# Supplementary material for: MicroRNA-621 Acts as a Tumor Radiosensitizer by Directly Targeting SETDB1 in Hepatocellular Carcinoma
Source: Mol Ther. 2018 Nov 13;27(2):355–64. doi: 10.1016/j.ymthe.2018.11.005 (PMC6369664; doi:10.1016/j.ymthe.2018.11.005)
Supplement: Document S1. Figures S1–S3 and Table S1 [file mmc1.pdf]

YMTHE, Volume *27*

Supplemental Information

MicroRNA-621 Acts as a Tumor Radiosensitizer by Directly Targeting SETDB1 in Hepatocellular Carcinoma

Yingjie Shao, Xing Song, Wenjie Jiang, Yuan Chen, Zhonghua Ning, Wendong Gu, and Jingting Jiang

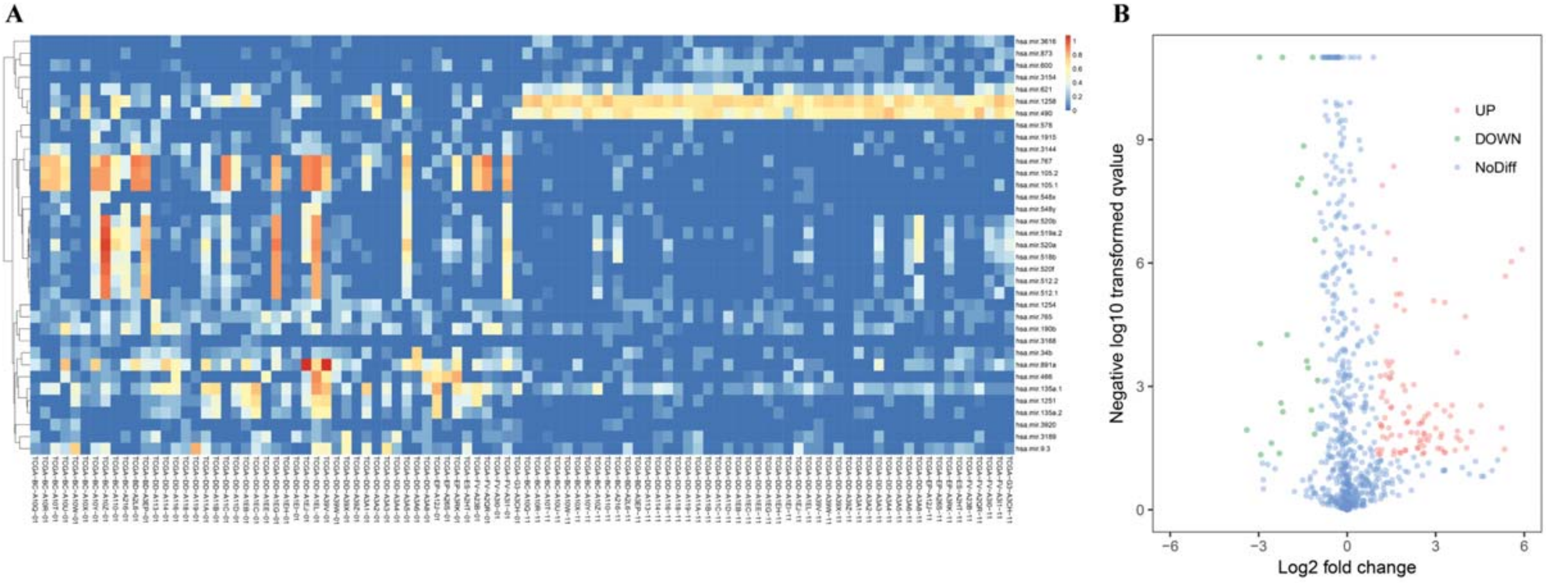

Supplemental Figure 1 Analysis of differentially expressed miRNAs in HCC by TCGA datasets (Fold change >3.0, P<0.01 in *t-test*). (A) Differentially expressed miRNAs shown in heatmap. (B) Differentially expressed miRNAs shown in volcano plot.

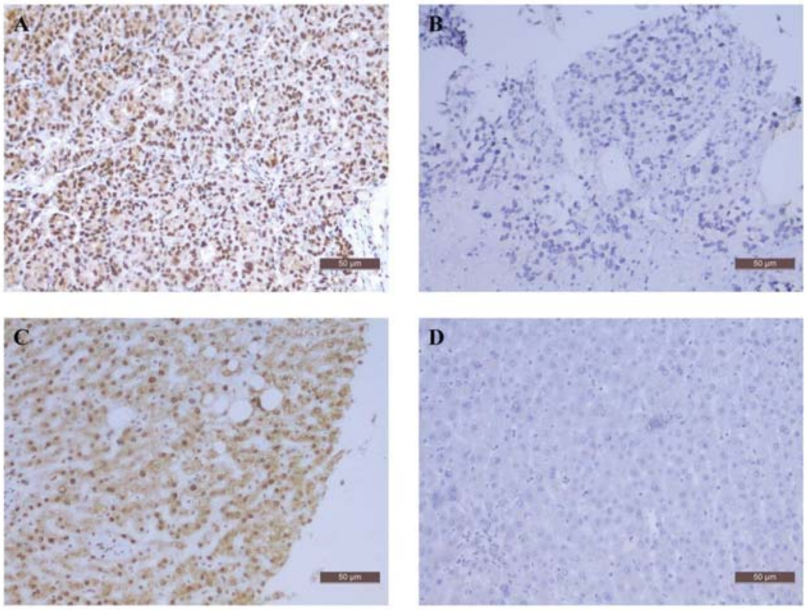

• **Supplemental Figure 2** SETDB1 IHC staining of (A) HCC tumor tissue with high SETDB1 expression and (B) low SETDB1 expression; (C) adjacent non-tumor tissue with high SETDB1 expression and (D) low SETDB1 expression.

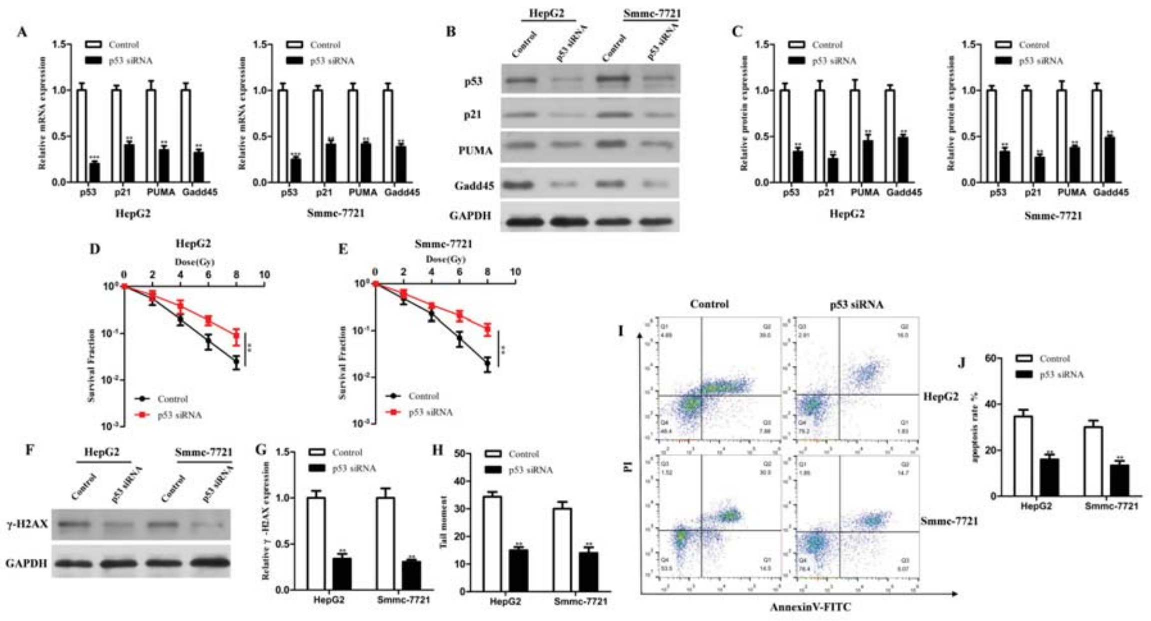

• **Supplemental Figure 3** Knock-down p53 enhances HCC cell radioresistance *in vitro*. (A) Expression of p53, p21, PUMA and Gadd45 mRNA detected by qRT-PCR in HepG2 and Smmc-7721 cells transfected with p53 siRNA or control. (B) Western blotting analysis of p53, p21, PUMA and Gadd45 protein in HepG2 and Smmc-7721 cells transfected with p53 siRNA or control. (C) Quantification of p53, p21, PUMA and Gadd45 protein. (D-E) HepG2 and Smmc-7721 cells transiently transfected with p53 siRNA or control were irradiated with a range of 2 to 8 Gy IR doses. After 14 days, colonies were stained and scored for colony formation and the SF was plotted as described in methods. (F) Western blotting analysis of γ-H2AX protein in HepG2 and Smmc-7721 cells transfected with p53 siRNA or control treated with a 4-Gy dose of IR. (G) Quantification of γ-H2AX protein. (H) The comet assay was used to evaluate DNA damage in HepG2 and Smmc-7721 cells transiently transfected p53 siRNA or control. Quantification of tail moment. (I-J) HepG2 and Smmc-7721 cells transfected with p53 siRNA or control were treated with a 4-Gy dose of IR. Treated cells were cultured for 24 h, harvested, stained with Annexin V-FITC/PI, and then analyzed using flow cytometry. Representative images and quantitative data are shown. Each column is shown as the means of three separate experiments. *P<0.05, **P<0.01, ***P<0.001.

Supplemental Table 1 Primer sequences used for qPCR assays

Gene	Sequence of the primers
SETDB1-Forward	5'-AAGACCAGAAGCTCCGTGAA-3'
SETDB1-Reverse	5'-CCTGGGAACTGCTCTTCTTG-3'
p21-Forward	5'-GCAGACCAGCATGACAGATTTC-3'
p21-Reverse	5'-ATGTAGAGCGGGCCTTTGAG-3'
PUMA-Forward	5'-CAGACTGTGAATCCTGTGCT-3'
PUMA-Reverse	5'-ACAGTATCTTACAGGCTGGG-3'
Gadd45-Forward	5'-TGCGAGAACGACATCAACAT-3'
Gadd45-Reverse	5'-TCCCGGCAAAAACAAATAAG-3'
p53-Forward	5'-GAGGTTGGCTCTGACTGTACC-3'
p53-Reverse	5'-TCCGTCCCAGTAGATTACCAC-3'
GAPDH-Forward	5'-CTCTCTGCTCTCCTGTTCGAC-3'
GAPDH-Reverse	5'-TGAGCGATGTGGCTCGGCT-3'
miR-621	5'-GGCTAGCAACAGCGCTTACCT-3'
U6	5'-CGCAAGGATGACACGCAAATTC-3'
