# Supplementary material for: MicroRNA-621 Acts as a Tumor Radiosensitizer by Directly Targeting SETDB1 in Hepatocellular Carcinoma
Source: Mol Ther. 2018 Nov 13;27(2):355–64. doi: 10.1016/j.ymthe.2018.11.005 (PMC6369664; doi:10.1016/j.ymthe.2018.11.005)
Supplement: Document S2. Article plus Supplemental Information [file mmc2.pdf]

Molecular Therapy
Original Article

MicroRNA-621 Acts as a Tumor Radiosensitizer by Directly Targeting SETDB1 in Hepatocellular Carcinoma

Yingjie Shao,[1] Xing Song,[1] Wenjie Jiang,[1] Yuan Chen,[1] Zhonghua Ning,[1] Wendong Gu,[1] and Jingting Jiang[2,3]

[1]Department of Radiation Oncology, The Third Affiliated Hospital of Soochow University, Changzhou 213003, Jiangsu Province, China; [2]Department of Tumor Biological Treatment, The Third Affiliated Hospital of Soochow University, Changzhou 213003, China; [3]Jiangsu Engineering Research Center for Tumor Immunotherapy, Changzhou 213003, China

Radiotherapy is one of the most important treatment methods of tumors. However, the application of radiotherapy in hepatocellular carcinoma (HCC) is limited due to the low tolerance of normal liver cells for radiation and inherent radiation resistance in HCC. With the in-depth study of microRNAs (miRNAs) in tumor therapy, the regulation of tumor radiosensitivity by miRNAs has been a research hotspot in recent years. In the present study, the expression of miR-621 was lower in HCC tissues and cells, and such low expression of miR-621 was associated with poor prognosis in HCC patients. In addition, *in vivo* and *in vitro* assays confirmed that the high expression of miR-621 could significantly enhance the radiosensitivity of HCC. Moreover, the expressions of miR-621 and SETDB1 in HCC tissues were negatively correlated. Dual-luciferase reporter assays indicated that miR-621 could directly target the 3′ UTR of SETDB1. In addition, miR-621 enhanced the radiosensitivity of HCC cells via directly inhibiting SETDB1. Besides, the miR-621 and/or SETDB1 axis improved the radiosensitivity of HCC cells via activating the p53-signaling pathway. Taken together, miR-621 and/or SETDB1 might be used as a novel therapeutic target for the treatment of HCC.

INTRODUCTION

Hepatocellular carcinoma (HCC) is the most common type of primary liver cancer in adults, which occurs in hepatic cells and the intrahepatic bile duct. As a type of severe disease, HCC seriously threatens human life and health. In 2012, there were 780,000 new cases of HCC and approximately 740,000 HCC-related deaths worldwide.[1] There is a high prevalence of HCC in China, where new cases and deaths of HCC account for more than half of its global sum.[2] At present, surgical treatment is the preferred therapeutic scheme for HCC. However, HCC is characterized by symptom-free onset, difficult early diagnosis, and rapid progression, and it is often complicated with liver cirrhosis. Clinically, only approximately 30% of HCC patients can obtain the opportunity for operative treatment, but the prognosis still remains poor.[3] Therefore, non-surgical treatment becomes an important alternative treatment means for advanced HCC. Although radiotherapy is one of the most important treatment methods of tumors, the application of radiotherapy in HCC is limited because of the low tolerance of normal liver cells for radiation and inherent radiation resistance in HCC.[4] Therefore, how to improve the radiosensitivity of HCC and protect the normal liver tissue from irradiation (IR) has become a current hot topic in the radiobiology and clinical radiotherapy of cancer.

Discovered in recent years, microRNAs (miRNAs) are a family of endogenous single-stranded non-coding RNA, approximately 21–25 nt in length, which are expressed in animals and plants.[5,6] With the in-depth study of miRNAs in tumor therapy, the regulation of tumor radiosensitivity via miRNAs has been a research hotspot in recent years.[7–11] miR-621 is located on chromosome 13q, and its deletion in liver cancer cells upregulates the cell-cycle-regulatory genes, leading to the proliferation of liver cancer cells.[12] In the present study, we found that miR-621 was one of the most significantly differentially expressed miRNAs between HCC and para-cancerous tissues. Meanwhile, we found that miR-621 could enhance the radiosensitivity of HepG2 and Smmc-7721.

The SET domain bifurcated 1 (SETDB1) gene is located on chromosome 1q21, which encodes a 143-kDa protein with multiple functional domains. SETDB1 possesses the activity of histone H3K9 methyltransferase, which mainly catalyzes the methylation of H3K9me3.[13] Its mechanism underlying such catalytic process may be that SETDB1 binds to HP1 and its co-repressor KAP1 in heterochromatin, thus promoting the formation of H3K9me3 and mediating the heterochromatin gene silencing.[14,15] It has been proven that SETDB1 is highly expressed in a variety of malignant tumors,[16–18] and it is closely related to the proliferation and invasion

Received 26 October 2018; accepted 7 November 2018;
https://doi.org/10.1016/j.ymthe.2018.11.005.

Correspondence: Wendong Gu, Department of Radiation Oncology, The Third Affiliated Hospital of Soochow University, 185 Juqian Street, Changzhou 213003, Jiangsu Province, China.
E-mail: guwendong1415@czfph.com
Correspondence: Jingting Jiang, Department of Tumor Biological Treatment, The Third Affiliated Hospital of Soochow University, Changzhou 213003, China.
E-mail: jiangjingting@suda.edu.cn

This is an open access article under the CC BY license (http://creativecommons.org/licenses/by/4.0/).

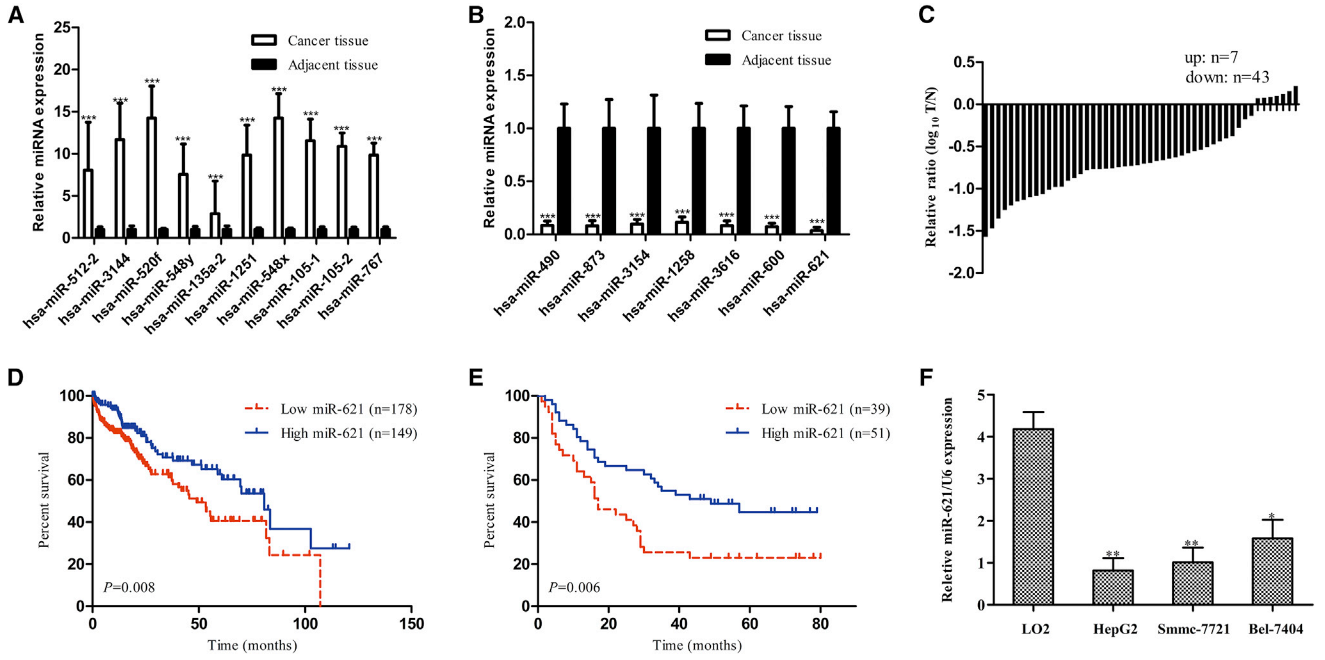

Figure 1. miR-621 Had Lower Expression in HCC Tissues and Cells, and a Low Expression of miR-621 Was Associated with Poor Survival in HCC

(A) Validation of the 10 most upregulated miRNAs in HCC from 20 HCC tumor and adjacent nontumor tissues was measured by real-time qPCR. (B) Validation of the seven most downregulated miRNAs in HCC from 20 HCC tumor and adjacent nontumor tissues was measured by real-time qPCR. (C) The expression of miR-621 in HCC tumor and matched nontumor tissues of 50 patients was detected using real-time qPCR. (D) Kaplan-Meier curve for TCGA datasets of 327 HCC patients. (E) Kaplan-Meier curve for an independent set of 90 HCC patients. (F) miR-621 expression in the human HCC lines HepG2, Smmc-7721, and Bel-7404 and the normal liver cell line LO2 was detected by real-time qPCR. *p < 0.05, **p < 0.01, ***p < 0.001.

of tumor cells. In HCC, SETDB1 is thought to be the epigenetic regulator that is upregulated most markedly, playing a key role in the proliferation and metastasis of HCC cells.[19,20] In the present study, it was found that SETDB1 was a direct target gene of miR-621, which could reverse the miR-621-induced radiosensitivity. Taken together, these findings suggested that miR-621 was a radiosensitizing miRNA, which might be used as a new therapeutic scheme for radioresistant tumors.

RESULTS

The Expression of miR-621 Is Decreased in HCC Tissues and Cells, and Such a Reduction of miR-621 Expression Is Associated with Poor Survival in HCC

To investigate the miRNA expression profile in HCC tissues, we analyzed 49 pairs of HCC and para-carcinoma tissues in The Cancer Genome Atlas (TCGA) database. The differential expression fold change >3 and p < 0.01 in t test in HCC and para-carcinoma tissues were used as the criteria, and we found that 28 miRNAs were upregulated and seven miRNAs were downregulated in HCC tissue (Figure S1). A total of the 10 most upregulated miRNAs (miR-512-2, miR-3144, miR-520f, miR-548y, miR-135a-2, miR-1251, miR-548x, miR-105-1, miR-105-2, and miR-767) and seven most downregulated miRNAs (miR-490, miR-873, miR-3154, miR-1258, miR-3616, miR-600, and miR-621) in HCC tissue were selected for further verification. The expressions of these miRNAs were verified in 20 pairs of HCC and para-carcinoma tissues by real-time qPCR, and we found that miR-621 was the most downregulated miRNA (Figures 1A and 1B).

Moreover, the expression of miR-621 in tumor and para-carcinoma tissues was examined in another 50 HCC patients. We showed that the expression of miR-621 in tumor tissue was obviously lower compared with the corresponding para-carcinoma tissue (Figure 1C). Furthermore, the correlation between miR-621 expression and prognosis of HCC patients was analyzed, and patients with low miR-621 expression had poor survival in TCGA datasets consisting of 327 HCC patients (p = 0.008; Figure 1D). The same result was verified in an independent set of 90 HCC patients who received operative treatment in our hospital from 2008 to 2010 (p = 0.007; Figure 1E). In addition, we detected the expression of miR-621 in LO2, HepG2, Smmc-7721, and Bel-7404 by real-time qPCR, and the result showed that the expression of miR-621 in HCC cell lines was significantly lower compared with the normal liver cell line (all p < 0.05; Figure 1F).

miR-621 Enhances the Radiosensitivity of HCC Cells *In Vitro*

To investigate the radiosensitivity of miR-621 in HCC, HepG2 and Smmc-7721 cells were transfected with miR-negative control (NC) or miR-621 mimic (Figures 2A and 2B). We found that a high expression of miR-621 could inhibit the survival fraction (SF) of HCC cells (Figures 2C and 2D). Apoptosis assay also confirmed that miR-621 could increase apoptosis of HCC cells upon IR (4 Gy) (Figures 2E

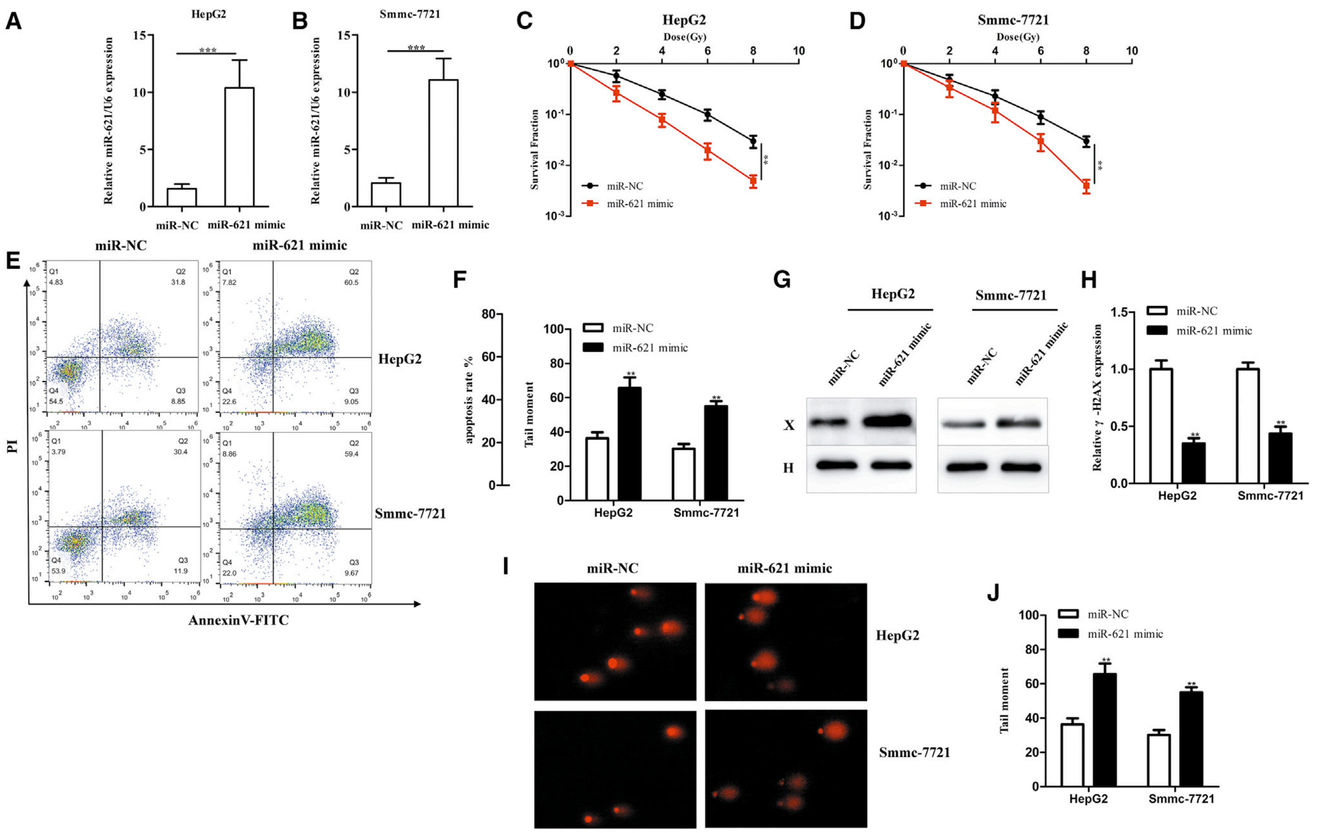

Figure 2. miR-621 Enhances HCC Cell Radiosensitivity *In Vitro*

(A and B) Expression of miR-621 was detected by real-time qPCR in HepG2 (A) and Smmc-7721 (B) cells transfected with miR-621 mimic or negative control (NC). (C and D) HepG2 (C) and Smmc-7721 (D) cells transiently transfected with miR-NC or miR-621 mimic were irradiated with a range of 2- to 8-Gy IR doses. After 14 days, colonies were stained and scored for colony formation and the SF was plotted, as described in the Materials and Methods. (E and F) Treated cells were cultured for 24 hr, harvested, stained with Annexin V-fluorescein isothiocyanate (FITC) and PI, and then analyzed using flow cytometry. Representative images (E) and quantitative data (F) are shown. Each column is shown as the means of three separate experiments. The late apoptotic cells are shown on the graph as PI+ Annexin V+ population. (G) Western blotting analysis of γ-H2AX protein in HepG2 and Smmc-7721 cells transfected with miR-621 mimic or miR-NC treated with a 4-Gy dose of IR. The expression of γ-H2AX protein was detected 6 hr after IR (4 Gy). (H) Quantification of γ-H2AX protein. (I) The comet assay was used to evaluate DNA damage in HepG2 and Smmc-7721 cells transiently transfected with miR-NC or miR-621 mimic. A representative cell nucleus fluorescent image is shown. (J) Quantification of tail moment. *p < 0.05, **p < 0.01, ***p < 0.001.

and 2F). In addition, overexpression of miR-621 in HCC cells triggered an increase of γ-H2AX (Figures 2G and 2H) after IR (4 Gy), an indicator of the cellular response to DNA damage. We also used a neutral comet assay to measure the levels of DNA damage. The comet assay showed the same result (Figures 2I and 2J). Therefore, miR-621 could increase the radiosensitivity of HCC.

SETDB1 Serves as a Direct Target Gene of miR-621

To investigate the potential target genes of miR-621, we conjointly analyzed the miRNA databases TargetScan (http://www.targetscan.org/), microRNA.org (http://www.microrna.org/microrna/home.do), and miRDB (http://www.mirdb.org/) and TCGA database. The most likely target genes (CLDN16, COQ2, LEF1, NGLY1, NLRP9, RBM11, UBQLN2, VAMP7, ZNF624, ALDH1A2, CDK8, EBF3, MIER1, NDNL2, PAK7, and SETDB1) were predicted through the three databases (Figure 3A). Then we analyzed 374 HCC tissues with the expression data of both these genes and miR-621 from TCGA database. The data showed that only four of them (LEF1, ZNF624, NLRP9, and SETDB1) were negatively correlated with miR-621 ($p < 0.05$). Moreover, the upregulation of miR-621 in HCC cells significantly decreased the expression of SETDB1 ($p < 0.001$; Figures 3B–3E). However, the expressions of other candidate target genes remained barely altered, suggesting that SETDB1 was a potential target gene of miR-621. The expressions of miR-621 and SETDB1 at the mRNA level in 367 HCC tissues were negatively correlated based on TCGA dataset ($r = -0.28$, $p < 0.001$; Figure 3F). The SETDB1 expression at the protein level in 50 pairs of HCC and adjacent normal tissues was detected by immunohistochemistry (IHC) staining (Figure S2). The expressions of miR-621 and SETDB1 protein in our independent set consisting of 50 HCC tissues were also negatively correlated ($r = -0.47$, $p = 0.001$; Figure 3G).

The mRNA and protein expressions of SETDB1 in HCC tissues were significantly lower compared with the para-carcinoma normal tissues

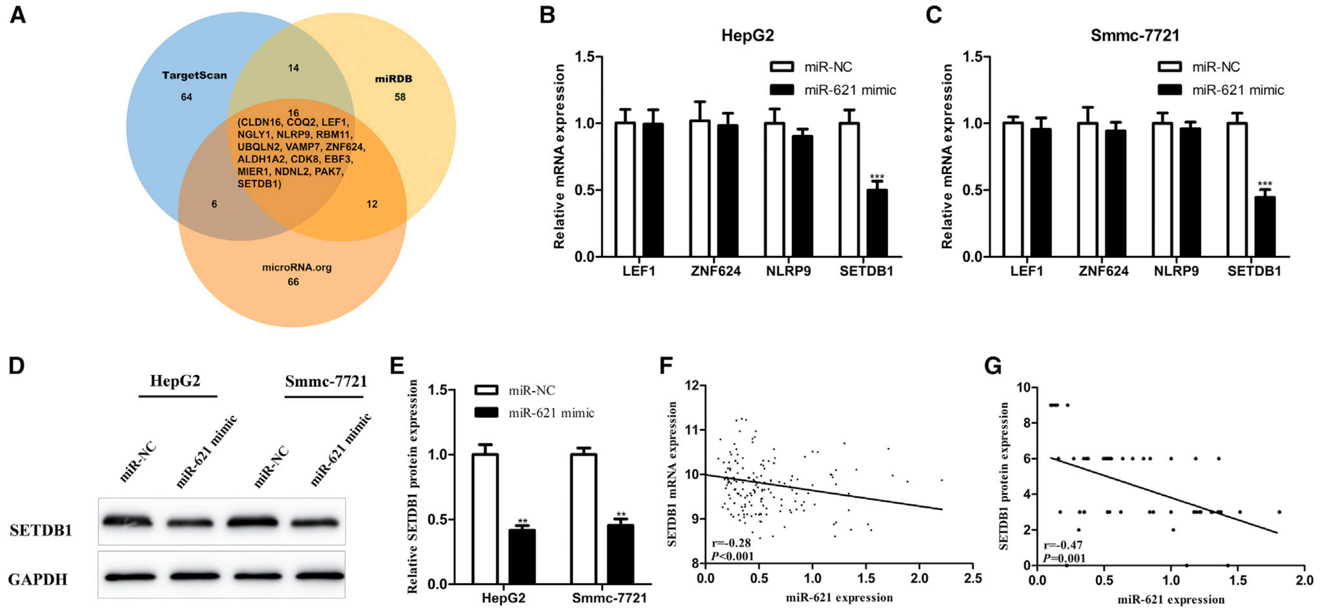

Figure 3. Screening and Validation of miR-637 Target Genes

(A) TargetScan, miRDB, and microRNA.org were used to predict target genes for miR-621. (B and C) Expressions of LEF1, ZNF624, NLRP9, and SETDB1 mRNA detected by real-time qPCR in HepG2 (B) and Smmc-7721 (C) cells transfected with miR-621 mimic or miR-NC. (D) Western blotting analysis of SETDB1 protein in HepG2 and Smmc-7721 cells transfected with miR-621 mimic or miR-NC. (E) Quantification of SETDB1 protein. (F) The expressions of miR-621 and SETDB1 mRNA in 367 HCC tissues were negatively correlated based on TCGA dataset ($r = -0.28$, $p < 0.001$). (G) The expression levels of miR-621 (detected by real-time qPCR) and SETDB1 protein (detected by IHC) in our independent set of 50 HCC tissues were also negatively correlated ($r = -0.47$, $p = 0.001$). *$p < 0.05$, **$p < 0.01$, ***$p < 0.001$.

($p < 0.001$; Figures 4A and 4B), and the expression of SETDB1 in HCC cells was also lower compared with the normal liver cells ($p < 0.001$; Figure 4C). Moreover, it was found that the upregulation of miR-621 in HCC cells significantly decreased the expression of SETDB1 at the mRNA and protein levels, indirectly proving that SETDB1 may be one of the target genes of miR-621.

We performed dual-luciferase reporter assays to verify whether SETDB1 was a direct target gene of miR-621. The miR-NC or miR-621 plasmid was co-transfected together with psiCheck2 or psiCheck2-SETDB1-wild-type (WT)/mutate (Mut) plasmid into HepG2 cell lines (Figure 4D). The luciferase assay showed that miR-621 had no obvious effects on SETDB1-Mut 3′ UTR, while the luciferase activity of the reporter plasmid inserted with SETDB-WT 3′ UTR was significantly decreased ($p = 0.005$). These findings confirmed that SETDB1 was a direct target gene of miR-621 (Figure 4E) and the binding site was the designated mutation site.

SETDB1 Reverses the Effects of miR-621 on Cell Radiosensitivity

To investigate whether the radiosensitivity of miR-621 to HCC cells was achieved via inhibiting SETDB1, we designed the following experiments. First, we examined whether SETDB1 can directly regulate the radiosensitivity of HCC cells. SETDB1 mRNA and protein were downregulated by transfection with small interfering RNA (siRNA) in HepG2 and Bel-7404 cells (Figures 5A–5C). Down-expression of SETDB1 could inhibit the SF of HCC cells (Figures 5D and 5E). In addition, down-expression of SETDB1 triggered an increase in DNA damage after IR (4 Gy) (Figures 5F–5H). Apoptosis assay also confirmed that down-expression of SETDB1 could increase the apoptosis of HCC cells (Figures 5I and 5J).

Second, HepG2 and Smmc-7721 cell lines were transfected under different conditions as follows: (1) miR-NC + vector control group, (2) miR-621 + vector control group, (3) miR-NC + SETDB1 without 3′ UTR group, and (4) miR-621 + SETDB1 without 3′ UTR group. The result showed that overexpression SETDB1 could completely reverse the effect of miR-621 on radiosensitivity of HCC cells (Figures 6A and 6B). Western blotting analysis and comet assay also showed that overexpression of SETDB1 could recover the miR-621-induced DNA damage after IR (Figures 6C–6E). In addition, apoptosis assay also confirmed that SETDB1 could reverse the miR-621-induced apoptosis (Figures 6F and 6G). In conclusion, miR-621 increased the radiosensitivity of HCC cells via directly inhibiting SETDB1.

miR-621 and/or SETDB1 Axis Enhances the Radiosensitivity of HCC Cells via Activating the p53-Signaling Pathway

Several studies have shown that SETDB1 is highly expressed in HCC and plays an oncogenic role in HCC. Fei et al.[20] have found that the high expression of SETDB1 in HCC is closely related to p53 expression. We investigated the relation between p53 activity and radiosensitivity in HCC cells. The expression of p53 and three important genes

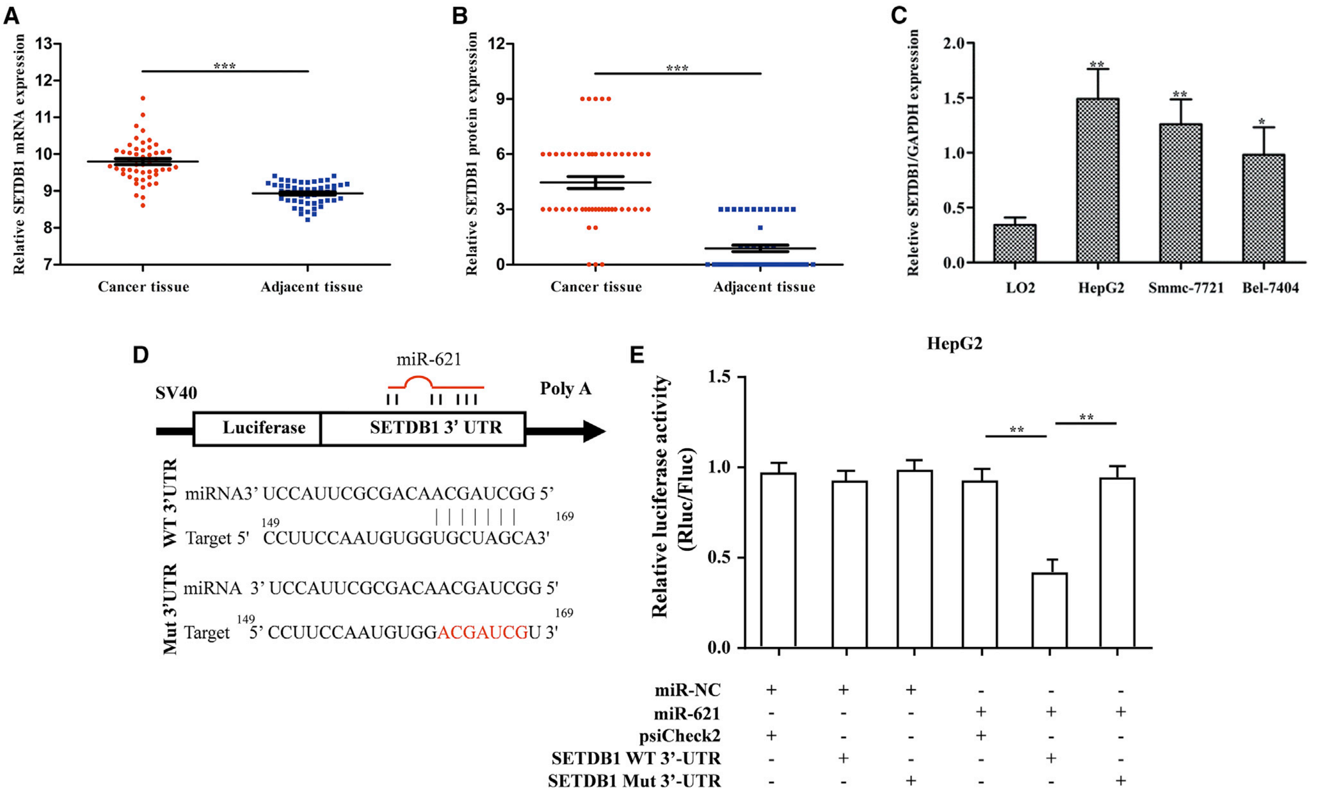

Figure 4. SETDB1 Serves as a Direct Target Gene of miR-621

(A) SETDB1 mRNA expression in 50 paired HCC tumor and adjacent nontumor tissues based on TCGA dataset. (B) SETDB1 protein expression (detected by IHC) in 50 paired HCC tumor and adjacent nontumor tissues based on our dataset. (C) SETDB1 expression in the human HCC lines HepG2, Smmc-7721, and Bel-7404 and the normal liver cell line LO2 was detected by real-time qPCR. (D) The construction of psiCheck2 report plasmid, containing WT or Mut SETDB1 3′ UTR. (E) The luciferase assay of HepG2 cells transfected with WT or Mut SETDB1 3′ UTR luciferase vectors and miR-621 mimic or miR-NC. The luciferase activity was measured at 24 hr after transfection. *$p < 0.05$, **$p < 0.01$, ***$p < 0.001$.

of p53 pathways (p21, PUMA, and Gadd45) was downregulated by transfection with siRNA in HepG2 and Bel-7404 cells (Figures S3A–S3C). It was found that knocked-down p53 induced cell radioresistance in HCC cells (Figures S3D–S3J).

To investigate whether the miR-621 and/or SETDB1 axis affected the p53-signaling pathway, HepG2 and Smmc-7721 cells were transfected with control, SETDB1 (without 3′ UTR), and/or miR-621 along with the p53 luciferase reporter. Subsequently, the transfected cells were exposed to a 4-Gy dose of IR. We found that overexpression of miR-621 could obviously activate p53-dependent transactivity, while SETDB1 could inhibit the p53-dependent transactivity induced by miR-621 (Figures 7A and 7B). In addition, we also detected the most important three effectors of p53 pathways (p21, PUMA, and Gadd45). It was found that the high expression of miR-621 could increase the expressions of p21, PUMA, and Gadd45 at the mRNA and protein levels, while overexpression of SETDB1 could reverse such upregulation of these factors (Figures 7C–7E). Collectively, miR-621 activated the p53 pathway via inhibiting SETDB1, eventually affecting the radiosensitivity of HCC cells.

miR-621 Enhances the Radiosensitivity of HCC *In Vivo*

The HCC mouse model was established based on a previously described method to investigate whether miR-621 could enhance the radiosensitivity in an animal model. When the tumor volume reached 200 mm^3, the tumor-bearing mice were randomly divided into four groups as follows: miR-NC agomir, miR-621 agomir, miR-NC agomir + IR, and miR-621 agomir + IR. The IR method and dosage are described in the Materials and Methods. Nude mice were executed by anesthesia at 30 days after IR, and the tumor volume was determined using a Vernier caliper. We found that the single injection of miR-621 agomir did not inhibit the tumor growth, while injecting miR-621 agomir into irradiated mice could significantly delay the tumor growth (Figures 8A and 8B). These results indicated that miR-621 could significantly increase the radiosensitivity *in vivo*. Moreover, we showed that the miR-621 expression of mice injected with miR-621 agomir was significantly higher compared with the control group, whereas the SETDB1 expression exhibited the opposite effect by real-time qPCR (Figure 8C). In addition, *in situ* hybridization (ISH) and IHC data clearly indicated that the expressions of miR-621 and SETDB1 were negatively correlated while the

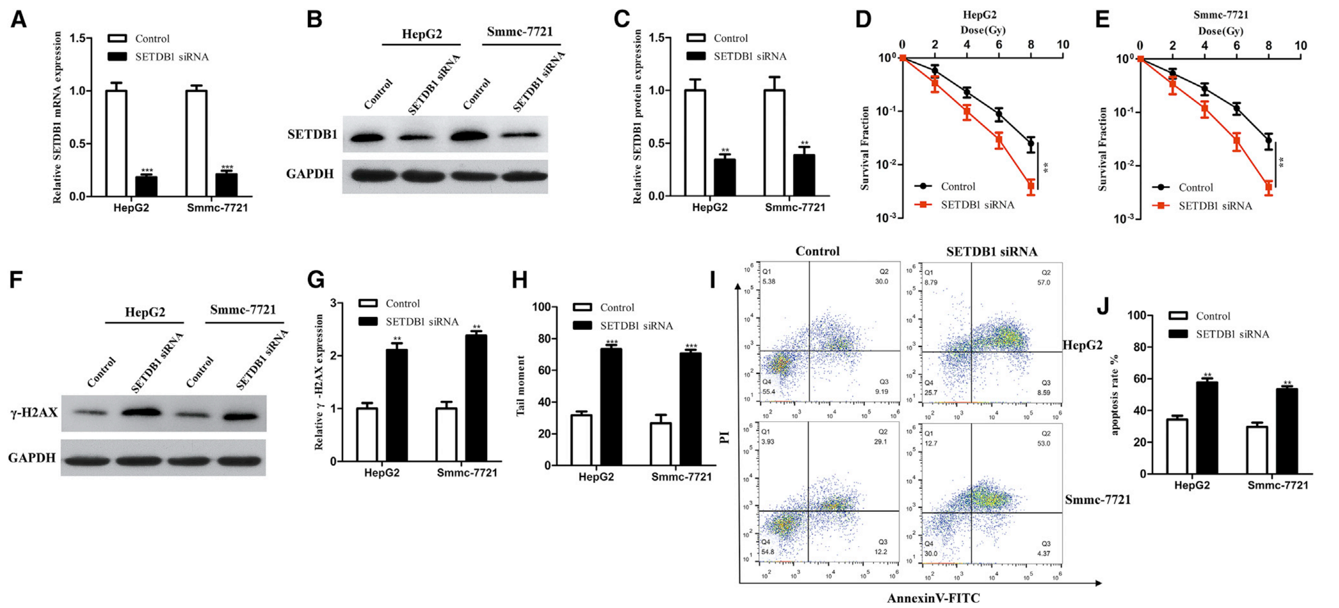

Figure 5. Down-Expression of SETDB1 Enhances HCC Cell Radiosensitivity *In Vitro*

(A) Expression of SETDB1 mRNA detected by real-time qPCR in HepG2 and Smmc-7721 cells transfected with SETDB1 siRNA or control. (B) Western blotting analysis of SETDB1 protein. (C) Quantification of SETDB1 protein. (D and E) HepG2 (D) and Smmc-7721 (E) cells transiently transfected with SETDB1 siRNA or control were irradiated with a range of 2- to 8-Gy IR doses. After 14 days, colonies were stained and scored for colony formation and the SF was plotted, as described in the Materials and Methods. (F) Western blotting analysis of γ-H2AX protein in HepG2 and Smmc-7721 cells transfected with SETDB1 siRNA or control treated with a 4 Gy dose of IR. (G) Quantification of γ-H2AX protein. (H) The comet assay was used to evaluate DNA damage in HepG2 and Smmc-7721 cells transiently transfected with SETDB1 siRNA or control. Quantification of tail moment is shown. (I and J) Treated cells were cultured for 24 hr, harvested, stained with Annexin V-FITC/PI, and then analyzed using flow cytometry. Representative images (I) and quantitative data (J) are shown. Each column is shown as the means of three separate experiments. $^{*}p < 0.05$, $^{**}p < 0.01$, $^{***}p < 0.001$.

expressions of miR-621 and p21, PUMA, and Gadd45 were positively correlated (Figure 8D). In summary, miR-621 could be used as a radiosensitizer, providing a new therapeutic approach for HCC treatment.

DISCUSSION

Radiotherapy is an important therapeutic scheme for tumors, and its effect is significant. However, HCC is not sensitive to radiotherapy, and the treatment is accompanied with severe side effects, undoubtedly limiting the development of radiotherapy in HCC. In radiotherapy, DNA damage is caused in cancer cells to different extents through ionizing radiation, and most cells can repair such damage automatically. However, the failure of DNA damage repair will lead to cell death directly or indirectly. Such damage repair ability affects the radiosensitivity of cells to a large extent. At present, studies have confirmed that miRNAs can regulate cell damage induced by ionizing radiation through various signal pathways at different levels. Therefore, the radiosensitivity of HCC was studied with miRNA as a breakthrough.

Previous studies have considered that miR-621 is a tumor suppressor gene and its deletion can lead to the upregulation of cell-cycle-regulatory genes, thus resulting in the proliferation of HCC cells.[12] Xue et al.[21] also indicated that overexpression miR-621 can inhibit FBXO11, ultimately enhancing chemosensitivity of breast cancer cells. In this study, our data showed that miR-621 had low expression in both HCC tissues and cell lines. Moreover, the prognosis of patients with a low expression of miR-621 was inferior to that of patients with a high expression of miR-621. In addition, overexpression of miR-621 could significantly enhance the radiosensitivity of HCC cells *in vivo* and *in vitro*. Furthermore, TCGA database and bioinformatics showed that the expression of miR-621 was significantly negatively correlated with SETDB1 and they had a binding site. Besides, dual-luciferase reporter assays in HepG2 cells confirmed that SETDB1 was a direct target gene of miR-621. Likewise, rescue assay was performed, and the results showed that, in HCC cell lines with miR-621 overexpression, the radiotherapy resistance of HCC cell lines was restored via the overexpression of SETDB1, suggesting that miR-621 enhanced the radiosensitivity of HCC cells via inhibiting SETDB1. Finally, it was verified that the miR-621 and/or SETDB1 axis could regulate the activity of the p53-signaling pathway.

SETDB1 is related to the transcriptional inhibition of euchromatin, which can maintain the function of embryonic liver cells via inhibiting the expression of specific genes. A great deal of evidence has proven that the differential expression of SETDB1 is closely related to tumors, and SETDB1 in TCGA database is among the top ones of the highly expressed genes in tumors. Wong et al.[19] found that

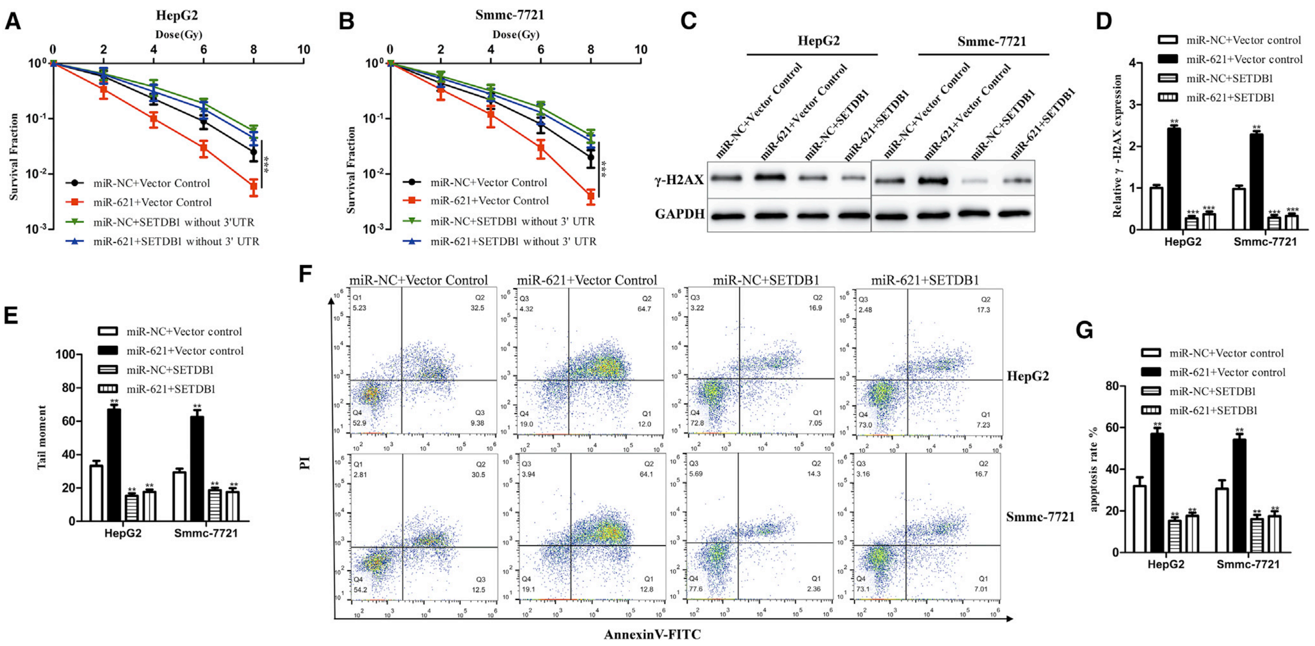

Figure 6. SETDB1 Reverses the Effects of miR-621 on Cell Radiosensitivity

HepG2 (A) and Smmc-7721 (B) cells transfected with miR-NC + vector control, miR-621 mimic + vector control, miR-NC + SETDB1 without 3′ UTR, or miR-621 mimic + SETDB1 without 3′ UTR were irradiated with a range of 2 Gy to 8 Gy IR doses. After 14 days, colonies were stained and scored for colony formation, and the SF was plotted as described in the Materials and Methods. (C) The expression of γ-H2AX protein was detected 6 h after IR by western blotting. (D) Quantification of γ-H2AX protein. (E) Quantification of tail moment. (F and G) Treated cells were cultured for 24 hr, harvested, stained with Annexin V-FITC/PI, and then analyzed using flow cytometry. Representative images (F) and quantitative data (G) are shown. Each column is shown as the means of three separate experiments. $^{*}p < 0.05$, $^{**}p < 0.01$, $^{***}p < 0.001$.

SETDB1 is the epigenetic regulator that is upregulated most significantly of known epigenetic regulators in HCC. The overexpression of SETDB1 was significantly related to HCC progression, cancer invasiveness, and poor survival of HCC patients. In particular, the inactivation of SETDB1 reduced the proliferation and migration capacities of HCC cells, indicating that SETDB1 was an important oncogene of HCC. Fei et al.[20] also found that SETDB1 is overexpressed in HCC and its copy number is increased moderately. SETDB1 can realize the dimethylation of p53K370. Lowly expressed SETDB1 decreases the p53K370me2 level, leading to increased recognition and degradation of p53 via MDM2 and, ultimately, affecting the HCC cell function. These experimental results are consistent with our findings.

In conclusion, miR-621 enhanced the radiosensitivity of HCC cells and activated the p53-signaling pathway via inhibiting the expression of SETDB1. Collectively, miR-621 could be used as a radiosensitizer in HCC, providing a new therapeutic scheme for other radioresistant tumors.

MATERIALS AND METHODS

Specimen Collection

A total of 90 patients, who received operative treatment at The Third Affiliated Hospital of Soochow University from 2008 to 2010, was selected in the present study. The collected tissue specimens were stored in liquid nitrogen at −80°C. The collection of clinical specimens was approved by the Ethics Committee of our hospital according to the Declaration of Helsinki. Every patient provided informed consent.

IHC

The tissues were microwave-treated for 2 hr and then boiled in 0.01 M citrate buffer (pH 6.0). Subsequently, the tissues were treated with 0.5% Triton for 20 min to break the cell membrane and then incubated with mouse antibody against human SETDB1 (1:300; Merck Millipore, Darmstadt, Germany). IHC evaluation and scoring were as described in our previous article.[22]

ISH

The expression of miR-621 in mouse tissue was assessed by ISH using a digoxigenin-labeled locked nucleic acid (LNA)-miR-621 probe (Exiqon, Vedbaek, Denmark). Nuclear fast red was used as the counterstain. For the NC, digoxigenin-labeled LNA-scrambled miRNA was used.

TCGA Datasets

To study the differential expression of mRNA and miRNA in HCC, the expression data of miRNA and mRNA were downloaded from HCC TCGA datasets using the UCSC cancer genome browser (https://xena.ucsc.edu/). The expression levels of miRNA and mRNA were determined by the Illumina HiSeq platform. A total of 327 HCC patients with both miR-621 expression and survival data were used in prognostic analysis, while 367 patients with both mRNA and miRNA expression data were employed in correlation analysis.

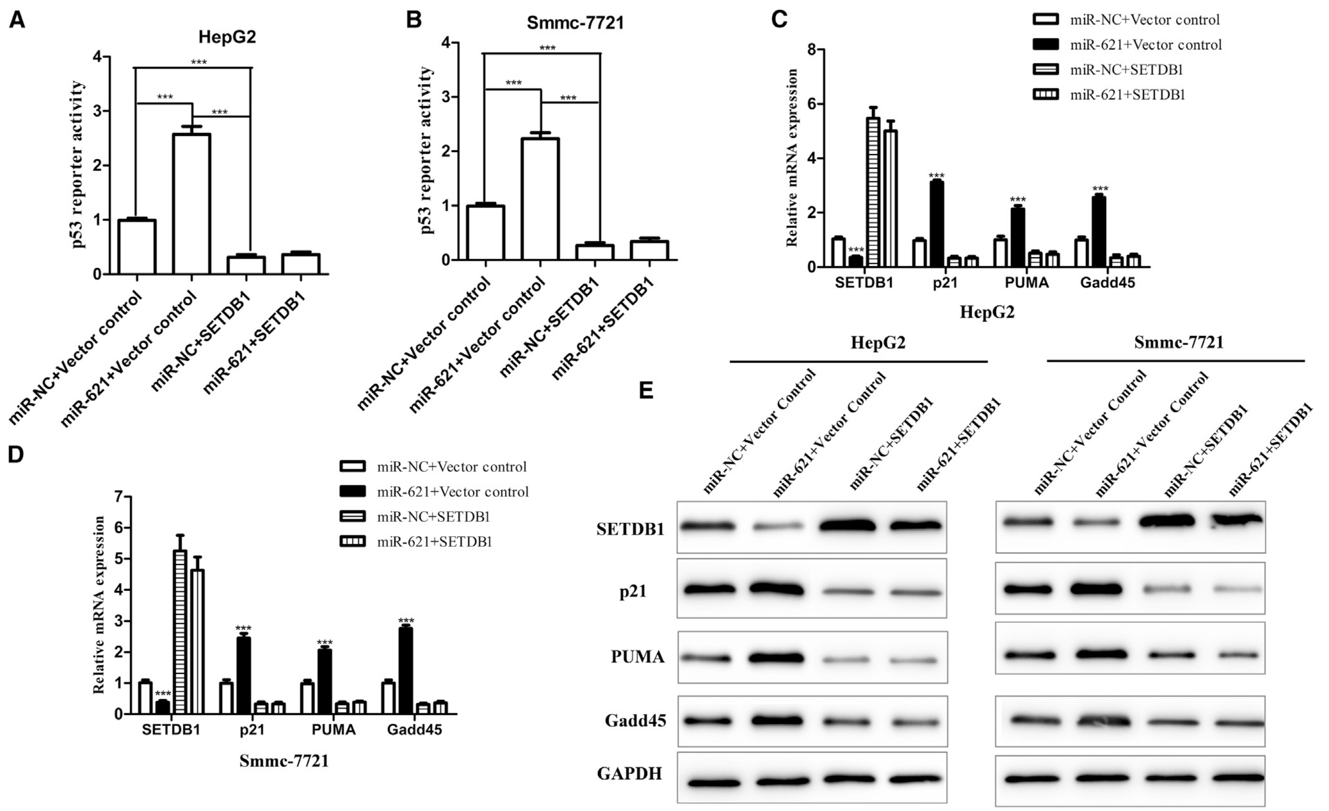

Figure 7. miR-621 and/or SETDB1 Axis Enhances the Radiosensitivity of HCC Cells by Activating the p53-Signaling Pathway

(A and B) HepG2 (A) and Smmc-7721 (B) cells transfected with miR-NC + vector control, miR-621 mimic + vector control, miR-NC + SETDB1 without 3′ UTR, or miR-621 mimic + SETDB1 without 3′ UTR along with the p53 luciferase reporter were treated with a 4-Gy dose of IR. The relative luciferase activities were evaluated 48 hr later using the Dual-Luciferase Reporter Assay Kit. (C and D) Expression of SETDB1 and the most important three effectors of p53 pathways (p21, PUMA, and Gadd45 mRNA) were detected by real-time qPCR: (C) HepG2 and (D) Smmc-7721. (E) Western blotting analysis of SETDB1, p21, PUMA, and Gadd45 protein in cells treated with a 4-Gy dose of IR. *$p < 0.05$, **$p < 0.01$, ***$p < 0.001$.

Moreover, HCC patients with both tumor and para-carcinoma tissues were adopted to examine the differential expressions of mRNA and miRNA.

Cell Lines, Plasmids, and Reagents

LO2, HepG2, Smmc-7721, and Bel-7404 cell lines were purchased from Shanghai Cell Bank of Chinese Academy of Sciences. Cell lines were authenticated by Genelily Biotechnology (Shanghai, China) using short tandem repeat analysis. The complete miR-621 gene was cloned into a pLenti (pLenti-CMV-GFP-puro)-cytomegalovirus (CMV)-GFP-puro vector (Clontech Laboratories, San Francisco, CA, USA). SETDB1 (lacking the 3′ UTR) expression constructs were subcloned into the pcDNA3.1 vector (Invitrogen, Carlsbad, CA, USA). In addition, miR-621 mimic, miR-NC mimic, SETDB1 siRNA, p53 siRNA, and NC were purchased from Thermo Fisher Scientific (Waltham, MA, USA).

Real-Time qPCR

Real-time qPCR analysis of miR-621 expression was reverse transcribed using specific reverse transcription (RT) primers (RiboBio, Guangzhou, China), while U6 was used as an internal control. The expressions of SETDB1, p21, PUMA, and Gadd45 at the mRNA level were assessed by real-time qPCR using specific RT primers (RiboBio, Guangzhou, China), and GAPDH was selected as a housekeeping gene. The expression levels of mRNA and miRNA were calculated using the comparative CT ($2^{-\Delta\Delta Ct}$) method. Table S1 gives all primer sequences.

Western Blotting Analysis

Western blotting analysis was as described in our previous article.[22] Briefly, 72 hr after transfection, cells were lysed with radioimmunoprecipitation assay (RIPA) buffer (Sigma-Aldrich, St. Louis, MO, USA), and then western blotting was carried out with standard procedures. The primary antibodies included SETDB1 antibody (1:1,000; Cell Signaling Technology, Boston, MA, USA), p53 antibody (1:1,000; Cell Signaling Technology, Boston, MA, USA), p21 antibody (1:1,000; Cell Signaling Technology, Boston, MA, USA), PUMA antibody (1:1,000; Cell Signaling Technology, Boston, MA, USA), Gadd45 antibody (1:1,000; Cell Signaling Technology, Boston, MA, USA), γ-H2AX antibody (1:1,000; Cell Signaling Technology, Boston, MA, USA), and GAPDH antibody (1:2,000; Santa Cruz

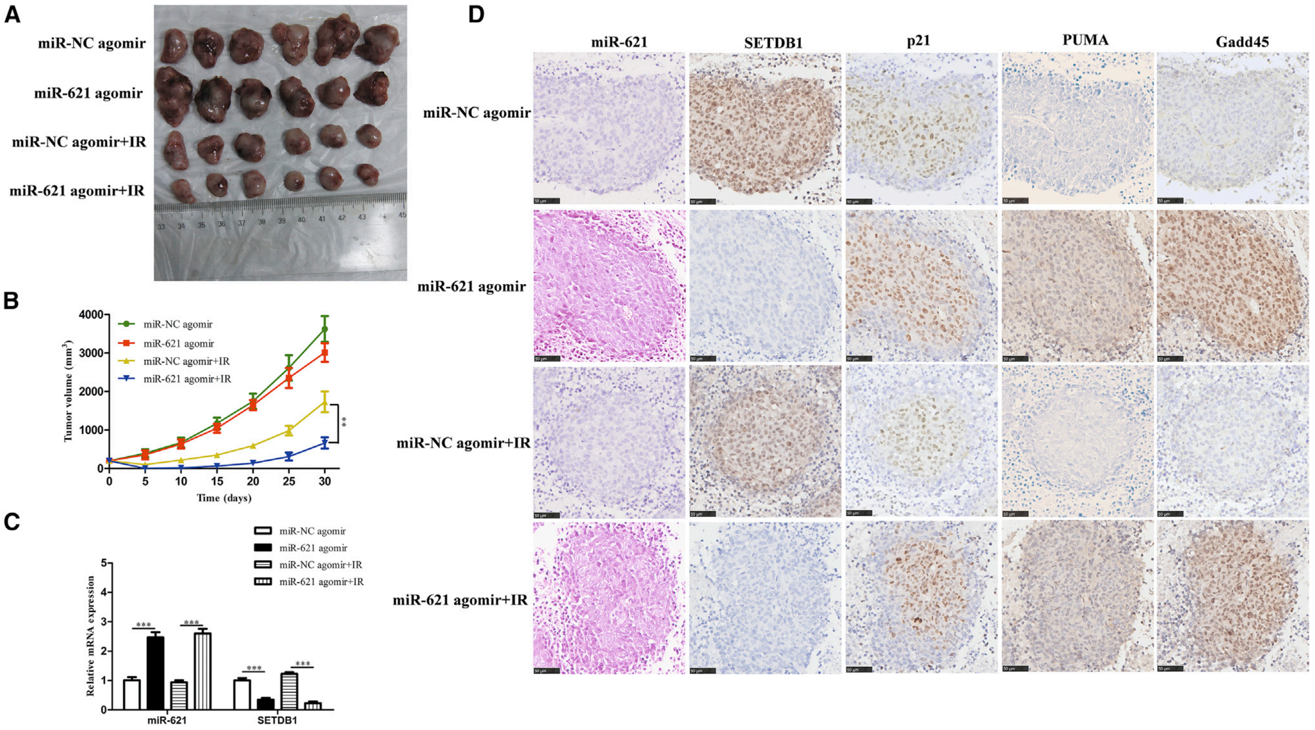

Figure 8. miR-621 Enhances the Radiosensitivity of HCC *In Vivo*

Mice were divided into the miR-NC agomir group, miR-621 agomir group, miR-NC agomir + IR group, and miR-621 agomir + IR group. (A) Tumors collected for weighing and analyses of gene expression. (B) Growth curves of four groups. (C) The miR-621 and SETDB1 mRNA expressions were measured by real-time qPCR. (D) miR-621 expression was detected via ISH. SETDB1, p21, PUMA, and Gadd45 protein expressions were detected via IHC. *p < 0.05, **p < 0.01, ***p < 0.001.

Biotechnology). The secondary antibodies included anti-mouse horseradish peroxidase (HRP)-conjugated antibody (1:5,000; Bio-Rad, Hercules, CA, USA) or anti-rabbit-HRP (1:5,000; Cell Signaling Technology, Boston, MA, USA).

Luciferase Reporter Assays

For dual-luciferase reporter assays, HepG2 cells were transfected with different combinations of miR-621, miR-NC, psiCHECK-2-SETDB1 3′ UTR-WT, and psiCHECK-2-SETDB1 3′ UTR-Mut for 24 hr. For p53 reporter activity, HepG2 cells were transfected with control, SETDB1 without 3′ UTR, and/or miR-621 along with the p53 luciferase reporter. The relative luciferase activities were evaluated 48 hr later using the Dual-Luciferase Reporter Assay Kit (Promega, Madison, WI, USA). Fold induction of the reporters was calculated based on the relative Firefly luciferase activity, normalized to Renilla luciferase as the transfection control.

IR, Colony Formation, and Apoptosis Assays

HepG2 and Smmc-7721 cells transfected with miR-621 and/or SETDB1 lacking 3′ UTR were seeded into 6-well plates. Cells were then exposed to different doses (0, 2, 4, 6, 8, and 10 Gy) of 6-MV X-ray and cultured for 10–14 days. The colonies with more than 50 cells were observed and counted. The SF of each group was calculated according to a previous study.[11] For apoptosis assays, cells were stained with antibodies against propidium iodide (PI) and annexin V 24 hr after IR (4 Gy). To detect the cellular response to DNA damage, γ-H2AX was detected 6 hr after 4-Gy IR.

Neutral Comet Assay

Neutral comet assay was performed to assess DNA damage in HCC cells using a CometAssay kit (R&D Systems, Minneapolis, MN, USA). The technique was performed according to the manufacturer's instructions. The slides were stained with ethidium bromide and photographed by fluorescence microscopy. The tail moment was quantified by CometScore 2.0 software (http://rexhoover.com/). 100 cells were analyzed for each group.

Animal Studies

All of the animal experiments were carried out according to the Regulations for the Administration of Affairs Concerning Experimental Animals. All animal-related experiments were approved and performed by The Third Affiliated Hospital of Soochow University. Briefly, 6-week-old mice were obtained from Soochow University. The HepG2 cells at a density of 1×10^6 were injected into the back of nude mice to establish the HCC model of tumor-bearing mice. When the tumor volume reached 200 mm^3, 24 tumor-bearing mice were evenly divided into 4 groups. Subsequently, 10 nmol miR-NC or miR-621 agomir (RiboBio, Guangzhou, China) in 50 μL saline

buffer was intratumorally injected into the tumor mass at multiple sites per mouse during 5 consecutive days. Next, a 10-Gy dose of IR was delivered to the tumor or not. Then, 10 nmol miR-NC or miR-621 agomir in 50 μL saline was injected into the tumor mass every 3 days. Based on the above-mentioned treatments, mice were divided into the miR-NC agomir group, miR-621 agomir group, miR-NC agomir + IR group, and miR-621 agomir + IR group. The tumor volume of nude mice was measured every 5 days. Nude mice were executed via anesthesia at 30 days after IR, and the final tumor volume was determined using a Vernier caliper. The expression levels of miR-621, SETDB1, p21, PUMA, and Gadd45 in tumor tissues were assayed via real-time qPCR, ISH, and IHC assays.

Statistical Analysis

All experiments were repeated at least three times. Data are presented as the mean ± SD. Student's t test (unpaired, two tailed) was used to assess the difference between two groups. Spearman's correlation analysis was employed to detect the correlation between miR-621 and SETDB1 in tissues. Prism (version 6.01) software (La Jolla, CA, USA) and SPSS 20.0 software (IBM, Armonk, NY, USA) were used for statistical analyses.

SUPPLEMENTAL INFORMATION

Supplemental Information includes three figures and one table and can be found with this article online at https://doi.org/10.1016/j.ymthe.2018.11.005.

AUTHOR CONTRIBUTIONS

Y.S., W.G., and J.J. conceived and designed the study and helped to draft the manuscript. X.S., W.J., and Y.C. performed the data collection. Z.N. performed the statistical analysis. All authors read and critically revised the manuscript for intellectual content and approved the final manuscript.

CONFLICTS OF INTEREST

The authors declare no competing interests.

ACKNOWLEDGMENTS

This work was supported by fundings from the National Science and Technology Support Program (2015BAI12B12), the National Natural Science Foundation of China (81301960, 31701111, and 81803036), Special Funds of Science and Technology of the People's Livelihood Construction Condition of Jiangsu Province (BL2014034), the Natural Science Foundation of Jiangsu Province (BK20180186), the Science and Technology Bureau Foundation Application Project of Changzhou (CJ20179047), and the Sanitation Bureau Guidance Project of Changzhou (WZ201605).

REFERENCES

1. Torre, L.A., Bray, F., Siegel, R.L., Ferlay, J., Lortet-Tieulent, J., and Jemal, A. (2015). Global cancer statistics, 2012. CA Cancer J. Clin. *65*, 87–108.
2. Chen, W., Zheng, R., Baade, P.D., Zhang, S., Zeng, H., Bray, F., Jemal, A., Yu, X.Q., and He, J. (2016). Cancer statistics in China, 2015. CA Cancer J. Clin. *66*, 115–132.
3. El-Serag, H.B. (2011). Hepatocellular carcinoma. N. Engl. J. Med. *365*, 1118–1127.
4. Tanguturi, S.K., Wo, J.Y., Zhu, A.X., Dawson, L.A., and Hong, T.S. (2014). Radiation therapy for liver tumors: ready for inclusion in guidelines? Oncologist *19*, 868–879.
5. Lewis, B.P., Burge, C.B., and Bartel, D.P. (2005). Conserved seed pairing, often flanked by adenosines, indicates that thousands of human genes are microRNA targets. Cell *120*, 15–20.
6. Krek, A., Grün, D., Poy, M.N., Wolf, R., Rosenberg, L., Epstein, E.J., MacMenamin, P., da Piedade, I., Gunsalus, K.C., Stoffel, M., and Rajewsky, N. (2005). Combinatorial microRNA target predictions. Nat. Genet. *37*, 495–500.
7. Gandellini, P., Rancati, T., Valdagni, R., and Zaffaroni, N. (2014). miRNAs in tumor radiation response: bystanders or participants? Trends Mol. Med. *20*, 529–539.
8. Zhang, Y., Zheng, L., Ding, Y., Li, Q., Wang, R., Liu, T., Sun, Q., Yang, H., Peng, S., Wang, W., and Chen, L. (2015). MiR-20a Induces Cell Radioresistance by Activating the PTEN/PI3K/Akt Signaling Pathway in Hepatocellular Carcinoma. Int. J. Radiat. Oncol. Biol. Phys. *92*, 1132–1140.
9. Cellini, F., Morganti, A.G., Genovesi, D., Silvestris, N., and Valentini, V. (2014). Role of microRNA in response to ionizing radiations: evidences and potential impact on clinical practice for radiotherapy. Molecules *19*, 5379–5401.
10. Qu, J.Q., Yi, H.M., Ye, X., Zhu, J.F., Yi, H., Li, L.N., Xiao, T., Yuan, L., Li, J.Y., Wang, Y.Y., et al. (2015). MiRNA-203 Reduces Nasopharyngeal Carcinoma Radioresistance by Targeting IL8/AKT Signaling. Mol. Cancer Ther. *14*, 2653–2664.
11. Zhang, P., Wang, L., Rodriguez-Aguayo, C., Yuan, Y., Debeb, B.G., Chen, D., Sun, Y., You, M.J., Liu, Y., Dean, D.C., et al. (2014). miR-205 acts as a tumour radiosensitizer by targeting ZEB1 and Ubc13. Nat. Commun. *5*, 5671.
12. Skawran, B., Steinemann, D., Becker, T., Buurman, R., Flik, J., Wiese, B., Flemming, P., Kreipe, H., Schlegelberger, B., and Wilkens, L. (2008). Loss of 13q is associated with genes involved in cell cycle and proliferation in dedifferentiated hepatocellular carcinoma. Mod. Pathol. *21*, 1479–1489.
13. Loyola, A., Tagami, H., Bonaldi, T., Roche, D., Quivy, J.P., Imhof, A., Nakatani, Y., Dent, S.Y., and Almouzni, G. (2009). The HP1alpha-CAF1-SetDB1-containing complex provides H3K9me1 for Suv39-mediated K9me3 in pericentric heterochromatin. EMBO Rep. *10*, 769–775.
14. Lundberg, L.E., Stenberg, P., and Larsson, J. (2013). HP1a, Su(var)3-9, SETDB1 and POF stimulate or repress gene expression depending on genomic position, gene length and expression pattern in Drosophila melanogaster. Nucleic Acids Res. *41*, 4481–4494.
15. Rowe, H.M., Friedli, M., Offner, S., Verp, S., Mesnard, D., Marquis, J., Aktas, T., and Trono, D. (2013). De novo DNA methylation of endogenous retroviruses is shaped by KRAB-ZFPs/KAP1 and ESET. Development *140*, 519–529.
16. Rodriguez-Paredes, M., Martinez de Paz, A., Simó-Riudalbas, L., Sayols, S., Moutinho, C., Moran, S., Villanueva, A., Vázquez-Cedeira, M., Lazo, P.A., Carneiro, F., et al. (2014). Gene amplification of the histone methyltransferase SETDB1 contributes to human lung tumorigenesis. Oncogene *33*, 2807–2813.
17. Ceol, C.J., Houvras, Y., Jane-Valbuena, J., Bilodeau, S., Orlando, D.A., Battisti, V., Fritsch, L., Lin, W.M., Hollmann, T.J., Ferré, F., et al. (2011). The histone methyltransferase SETDB1 is recurrently amplified in melanoma and accelerates its onset. Nature *471*, 513–517.
18. Chen, K., Zhang, F., Ding, J., Liang, Y., Zhan, Z., Zhan, Y., Chen, L.H., and Ding, Y. (2017). Histone Methyltransferase SETDB1 Promotes the Progression of Colorectal Cancer by Inhibiting the Expression of TP53. J. Cancer *8*, 3318–3330.
19. Wong, C.M., Wei, L., Law, C.T., Ho, D.W., Tsang, F.H., Au, S.L., Sze, K.M., Lee, J.M., Wong, C.C., and Ng, I.O. (2016). Up-regulation of histone methyltransferase SETDB1 by multiple mechanisms in hepatocellular carcinoma promotes cancer metastasis. Hepatology *63*, 474–487.
20. Fei, Q., Shang, K., Zhang, J., Chuai, S., Kong, D., Zhou, T., Fu, S., Liang, Y., Li, C., Chen, Z., et al. (2015). Histone methyltransferase SETDB1 regulates liver cancer cell growth through methylation of p53. Nat. Commun. *6*, 8651.
21. Xue, J., Chi, Y., Chen, Y., Huang, S., Ye, X., Niu, J., Wang, W., Pfeffer, L.M., Shao, Z.M., Wu, Z.H., and Wu, J. (2016). MiRNA-621 sensitizes breast cancer to chemotherapy by suppressing FBXO11 and enhancing p53 activity. Oncogene *35*, 448–458.
22. Shao, Y., Zhang, D., Li, X., Yang, J., Chen, L., Ning, Z., Xu, Y., Deng, G., Tao, M., Zhu, Y., and Jiang, J. (2018). MicroRNA-203 Increases Cell Radiosensitivity via Directly Targeting Bmi-1 in Hepatocellular Carcinoma. Mol. Pharm. *15*, 3205–3215.

YMTHE, Volume *27*

Supplemental Information

MicroRNA-621 Acts as a Tumor Radiosensitizer by Directly Targeting SETDB1 in Hepatocellular Carcinoma

Yingjie Shao, Xing Song, Wenjie Jiang, Yuan Chen, Zhonghua Ning, Wendong Gu, and Jingting Jiang

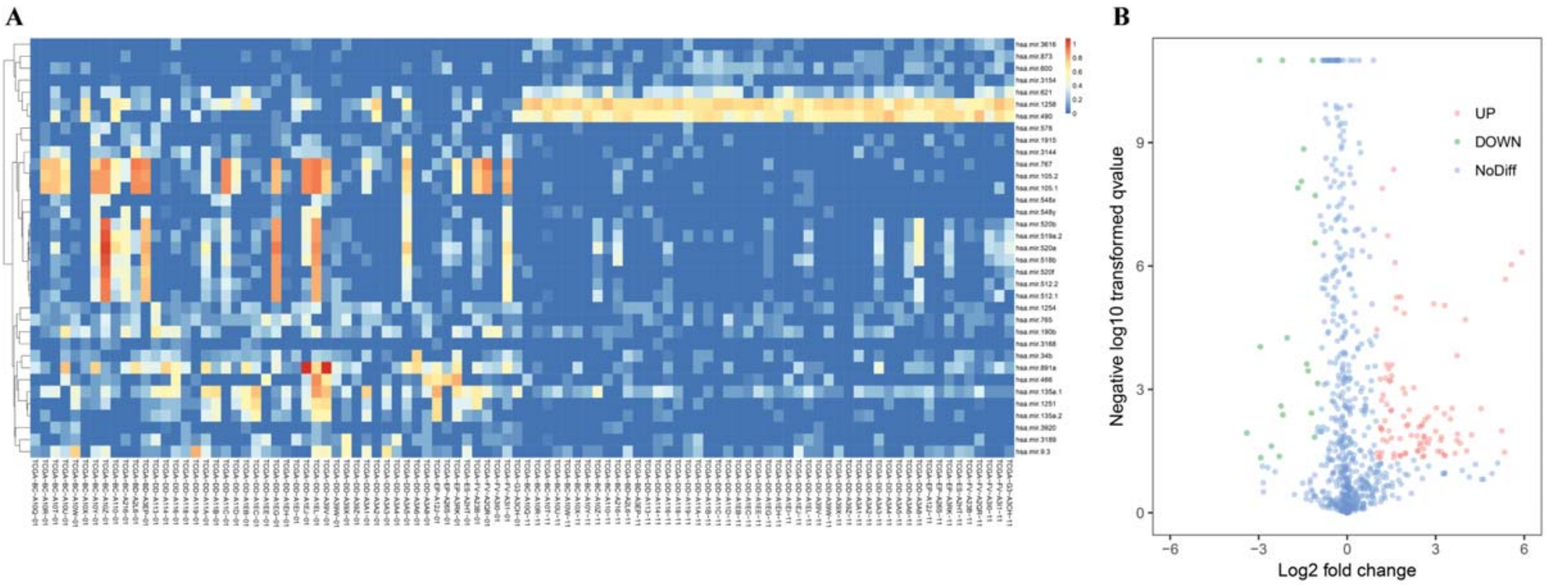

Supplemental Figure 1 Analysis of differentially expressed miRNAs in HCC by TCGA datasets (Fold change >3.0, *P*<0.01 in *t-test*). (A) Differentially expressed miRNAs shown in heatmap. (B) Differentially expressed miRNAs shown in volcano plot.

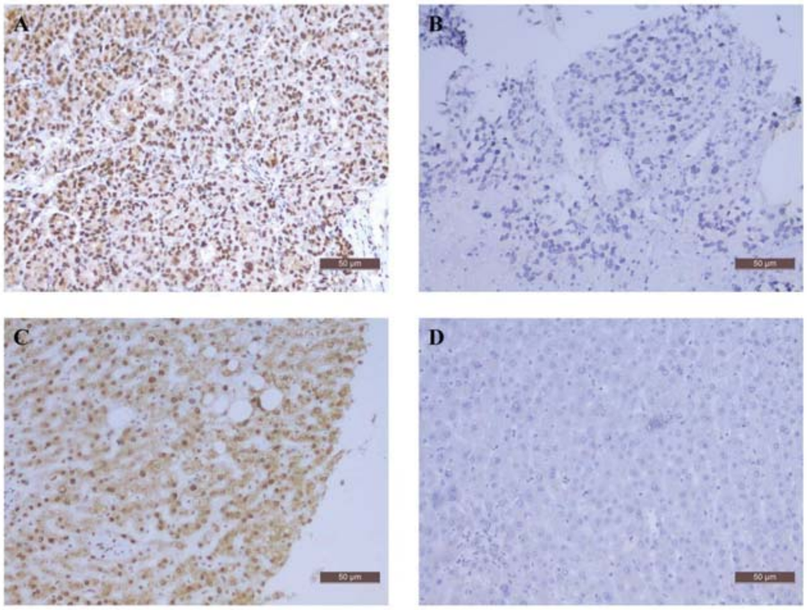

- **Supplemental Figure 2** SETDB1 IHC staining of (A) HCC tumor tissue with high SETDB1 expression and (B) low SETDB1 expression; (C) adjacent non-tumor tissue with high SETDB1 expression and (D) low SETDB1 expression.

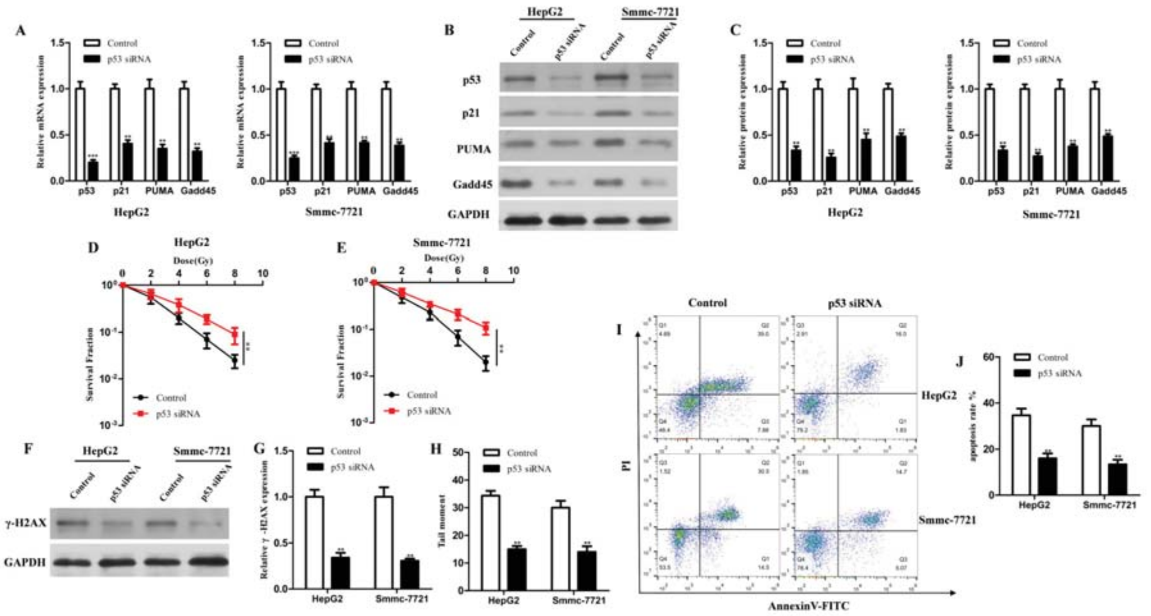

• **Supplemental Figure 3** Knock-down p53 enhances HCC cell radioresistance *in vitro*. (A) Expression of p53, p21, PUMA and Gadd45 mRNA detected by qRT-PCR in HepG2 and Smmc-7721 cells transfected with p53 siRNA or control. (B) Western blotting analysis of p53, p21, PUMA and Gadd45 protein in HepG2 and Smmc-7721 cells transfected with p53 siRNA or control. (C) Quantification of p53, p21, PUMA and Gadd45 protein. (D-E) HepG2 and Smmc-7721 cells transiently transfected with p53 siRNA or control were irradiated with a range of 2 to 8 Gy IR doses. After 14 days, colonies were stained and scored for colony formation and the SF was plotted as described in methods. (F) Western blotting analysis of γ-H2AX protein in HepG2 and Smmc-7721 cells transfected with p53 siRNA or control treated with a 4-Gy dose of IR. (G) Quantification of γ-H2AX protein. (H) The comet assay was used to evaluate DNA damage in HepG2 and Smmc-7721 cells transiently transfected p53 siRNA or control. Quantification of tail moment. (I-J) HepG2 and Smmc-7721 cells transfected with p53 siRNA or control were treated with a 4-Gy dose of IR. Treated cells were cultured for 24 h, harvested, stained with Annexin V-FITC/PI, and then analyzed using flow cytometry. Representative images and quantitative data are shown. Each column is shown as the means of three separate experiments. **P*<0.05, ***P*<0.01, ****P*<0.001.

Supplemental Table 1 Primer sequences used for qPCR assays

Gene	Sequence of the primers
SETDB1-Forward	5'-AAGACCAGAAGCTCCGTGAA-3'
SETDB1-Reverse	5'-CCTGGGAACTGCTCTTCTTG-3'
p21-Forward	5'-GCAGACCAGCATGACAGATTTC-3'
p21-Reverse	5'-ATGTAGAGCGGGCCTTTGAG-3'
PUMA-Forward	5'-CAGACTGTGAATCCTGTGCT-3'
PUMA-Reverse	5'-ACAGTATCTTACAGGCTGGG-3'
Gadd45-Forward	5'-TGCGAGAACGACATCAACAT-3'
Gadd45-Reverse	5'-TCCCGGCAAAAACAAATAAG-3'
p53-Forward	5'-GAGGTTGGCTCTGACTGTACC-3'
p53-Reverse	5'-TCCGTCCCAGTAGATTACCAC-3'
GAPDH-Forward	5'-CTCTCTGCTCTCCTGTTCGAC-3'
GAPDH-Reverse	5'-TGAGCGATGTGGCTCGGCT-3'
miR-621	5'-GGCTAGCAACAGCGCTTACCT-3'
U6	5'-CGCAAGGATGACACGCAAATTC-3'
